# Treatment of failed scaphoid nonunion fixation using free medial femoral condyle vascularized bone grafting

**DOI:** 10.1051/sicotj/2023004

**Published:** 2023-04-07

**Authors:** Islam Koriem, Aly Abdalla Agina, Ahmed K El Ghazawy

**Affiliations:** 1 Lecturer of Orthopedic Surgery, Ain Shams University 11566 Cairo Egypt; 2 Orthopedic Consultant Electricity Hospital, Ain Shams University 11566 Cairo Egypt

**Keywords:** Scaphoid, Non-Union, Vascularised bone grafts, medial femoral condyle, autografts

## Abstract

*Background:* Nonunion in scaphoid fractures may be considered a devastating problem. Union failure results in scaphoid deformity, resorption, and bone loss. Failed previous fixation decreases remaining bone stock and makes it more difficult to achieve union. Free vascularized graft represents a good option to achieve scaphoid union with revision fixation. Our study aims at the assessment of the management of scaphoid fractures non-union after failed previous fixation with the use of a free vascularized graft from the medial femoral condyle. *Methods:* This is a retrospective study including 16 cases with persistent scaphoid nonunion after previous fixation managed by vascularized medial femoral condyle grafts. The mean follow-up was 24 months. Previous surgical attempts and nonunion duration were noted. We evaluated the union rate, together with ROM, Scapholunate angles and pain scores. *Results:* the union was achieved in 13 of 16 cases. Pain improved in all patients (10/16 complete relief). Wrist ROM at follow-up was an average of 50° ﬂexion 48° extension. There was no change in the relationship between lunate and scaphoid with an average angle of 37.5° preoperative and 38° postoperative. *Conclusion:* Free vascularized MFC grafts are considered a reliable method to treat persistent nonunion of scaphoid fractures after failed previous operations. Short-term follow-up data showed considerable union rates with adequate pain relief and satisfactory ROM.

## Introduction

Scaphoid fractures account for 60% of carpal bone fractures and nonunion rates may reach up to 15% [1].

Degree of fracture displacement together with proximal fractures and scaphoid avascular necrosis (AVN) are considered risks for nonunion. Also, the presence of carpal instability or delayed cast immobilization may lead to nonunion as well [2].

Failure of achievement of the union after surgical intervention results in loss of available bone stock with long-term complications in the form of scaphoid deformity. The use of large-sized screws adds to bone stock resorption, with the risk of articular injury in case of implant migration. If a local vascularized grafting has been used with the fixation method the achievement of union may be a discarded idea and thoughts will be directed toward limited carpal fusions [3].

The free vascularized graft may represent a good option in persistent scaphoid nonunion, especially in those who show no signs of SNAC in X-rays and Ct or are accompanied by AVN [3, 4].

Grafts from the MFC represent an appropriate method in the management of failed scaphoid fixation especially those with an avascular proximal pole [5].

A medial femoral condyle graft is a corticoperiosteal pedicled graft the vascularity of which is obtained from descending genicular artery (DGA) and vein articular branch or superomedial genicular vessels [5]. This technique gained increased interest due to its rich vascular anatomy [6]. MFC graft can provide adequate structural support when used volarly with the improvement of union rates and scapholunate angles [7].

Current literature provides limited data regarding the results of vascularized grafts from the MFC in the management of non-united scaphoid fractures after failed previous fixation. This study aims at evaluating the short-term results and union rates of free vascularized MFC grafting as a treatment option for persistent scaphoid nonunions after failed previous surgical attempts.

## Materials and methods

This study is a retrospective series including 16 patients (6 females and 10 males) with recalcitrant nonunions of scaphoid fractures treated with free MFC vascularized grafting over 3 years (from 2019 to 2022) at Ain shams university hospitals. Follow-up was mean 24 months (range 6–30). The mean age was 37.4 yrs (27–49 yrs). The time since the last surgical fixation attempt was 1 yr (0–3yrs). Any patient with SNAC was excluded.

For preoperative evaluation, medical and surgical history together with local clinical examination were obtained from all patients, and data was collected about previous surgical interventions. Plain X-rays of the affected wrist were done in three different views (PA-LAT-Scaphoid) CT scan and MRI were done if needed. After the tourniquet release, the absence of punctuated bleeding of the scaphoid bone confirmed the AVN diagnosis.

*Surgical technique*: All patients received general anesthesia and were positioned supine with the operated limb placed on the side table. At the same time, we prepared ipsilateral L.L. from the thigh distally. Ipsilateral L.L. grafts are preferred to allow working simultaneously on both U.L. and L.L., also this makes it easier to use walking aids with the contralateral limb postoperatively. The operation was done under a tourniquet.

The extended anterior Russe approach was used for scaphoid exposure, graft application and vascular anastomosis. Incision made over flexor carpi radialis (FCR) tendon extending 8 cm proximally with mild radial curve distal to the wrist crease towards trapezium. The sheath of FCR was dissected with tendon retraction ulnarly, fascia was dissected longitudinally to expose the radiocarpal capsule over the scaphoid bone. The scaphoid fracture site was exposed with the removal of any retained implants. Refreshing of the nonunion site was done with the removal of any fibrous or necrotic tissues followed by an evaluation of the size of the present defect.

The femoral graft is then harvested from the ipsilateral lower limb. Under tourniquet using a distal thigh medial approach, starting from the medial joint line and extending 20 cm proximally. Deep fascia was dissected with retraction of the vastus medialis muscle anteriorly exposing the medial femoral condyle with an exploration of the DGA branching from SFA or the medial superior genicular artery branching from the popliteal artery. The vascular pedicle of chosen artery and vein was dissected in sufficient length. The bone graft of adequate size is then harvested with the division of the explored pedicle.

**Figure 1:**
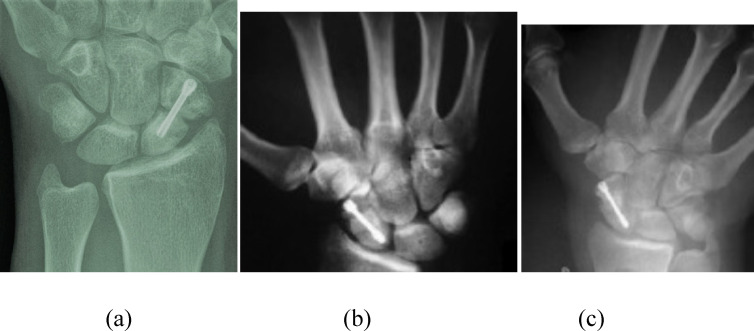
a) Preoperative PA wrist X-ray of non-united scaphoid fracture after fixation with Herbert screw. b) Postoperative X-ray after revision fixation using vascularized MFC graft and refixation. c) Follow-up X-ray showing full scaphoid union.

**Figure 2:**
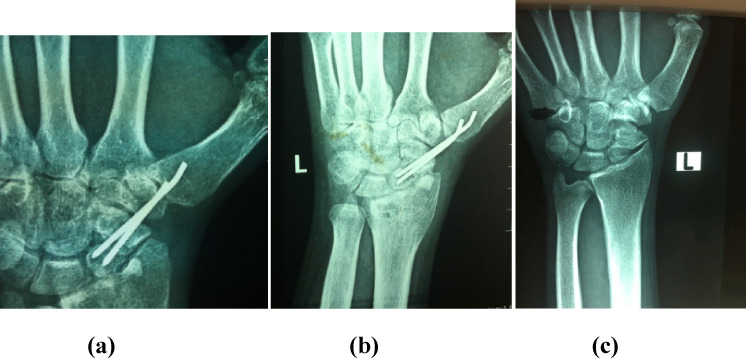
a) Postoperative PA wrist X-ray of revision fixation of non-united scaphoid using k wires and vascularized MFC graft. b) Follow-up X-ray. c) X-ray after wires removal showing full scaphoid union.

**Figure 3:**
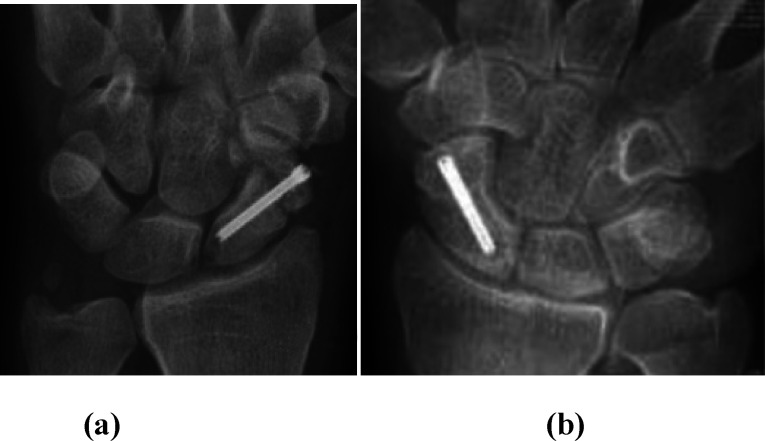
a) Preoperative PA wrist X-ray of no-nunited scaphoid fixed with Herbert screw. b) Follow-up X-ray showing full union after revision fixation using MFC graft.

The recipient site is then prepared with exposure of the radial artery and its vena comitans, followed by graft application after its adjustment according to defect morphology with fixation using screws or k-wires (Figures 1–3).

**Figure 4:**
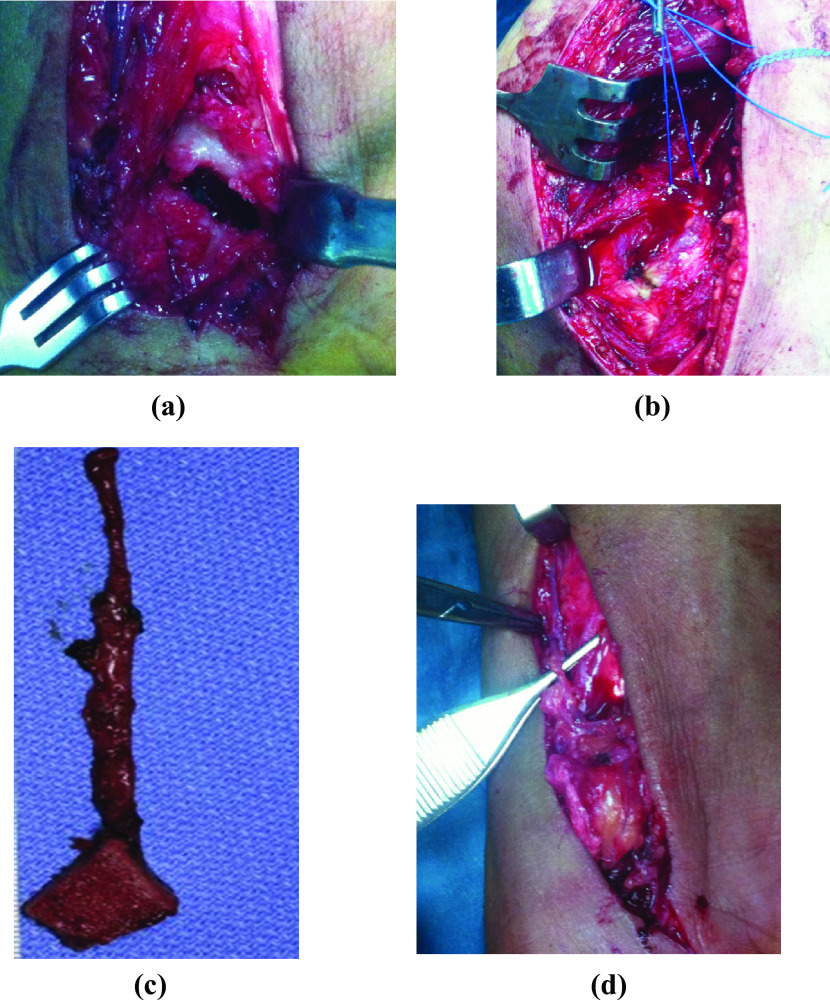
Intraoperative images, a) non-united scaphoid fracture site; b) harvesting vascularised MFC graft; c) graft with vascular pedicle; d) graft after vascular anastomosis.

The vascular anastomosis was done using surgical magnifying glasses (Loupes) to the radial artery using end-to-side or end-to-end technique into the radial artery palmar branch using 9-0 sutures. A similar anastomosis technique was used for venous anastomosis to a cephalic vein or vena comitans. Adequacy of vascular flow was then confirmed before wound closure with the use of a suction drain. Finally, the slab was done over the thumb in a neutral position (Figure 4).

Postoperatively thumb spica was kept for 6 weeks. Patients came for follow-up at 2, 4, 6, 8 weeks postoperatively and every month after that. Follow-up was mean 24 months (6–30 months). Stitches were removed after 2 weeks. Any wound complications or donor site morbidities were recorded.

Patients were assessed for scaphoid union by X-rays at every follow-up visit till the end of follow-up and confirmed by CT scan when needed. Interscaphoid and scapholunate angles were measured.

We recorded wrist ROM and grip strength. The pain was evaluated using a VAS score. Functional outcome was evaluated using the Mayo score. The patient’s functional satisfaction and working ability were recorded.

Regarding antibiotics protocol, all patients received a loading AB dose of 1st generation cephalosporins which was continued for 48 h postoperatively***.***

## Results

Union was confirmed radiologically in 13 of the 16 reconstructed scaphoids (82%). The two cases that showed non-union had risk factors including heavy smoking and follow-up noncompliance with DM in one of them. The implant position was maintained in all cases till the last follow-up (Table 1).

**Table 1:** Preoperative and postoperative characteristics of patients in whom scaphoid nonunion failed to unite after mfc bone-grafting.

Case	Age	Sex (yr)	Smoker?	Prior bone graft	Prior fixation	Method of fixation
1	40	Male	Yes	No	Screw	K wires
2	36	Male	Yes	Yes	K wires	K wires
3	24	Male	Yes	Yes	Screw	Screw

Considering functional evaluation VAS score improved from a mean of 6 preoperatively (from 3 to 9, SD = 1.9) to a mean of 2 postoperatively (from 0 to 4, SD = 1.6). This was statistically highly significant.

The final postoperative ROM: Flexion starting from neutral to a range from 38° to 72° with a mean of 52.31°, Extension starting from neutral to a range from 25° to 68° with a mean of 48.56°, Ulnar deviation starting from neutral to a range from 11° to 31° with a mean of 22.44° and Radial deviation starting from neutral to a range from 8° to 20° with a mean of 14.94°.

*Mayo score*: The preoperative score ranged from 10 to 70 with a mean of 45.63, and the final postoperative score ranged from 35 to 100 with a mean of 73.44. This improvement was statistically highly significant.

## Complications

Complications were scaphoid non-union in three patients (Table 1), Saphenous nerve paresthesia in 2 patients (12.5%)**,** postoperative donor site pain in 10 patients (62.5%), anterior knee pain in 5 patients (31.25%), radioscaphoid osteoarthritis in 2 patients and scaphoid proximal pole AVN in one patient (6.25%).

## Discussion

Non-union of scaphoid fractures leads to several complications including degeneration of the wrist joint which is directly related to the degree of fracture displacement and the presence of associated carpal instability. Persistence of non-union after previous fixation attempts leads to a reduction of remaining bone stock and its quality, making further corrective surgery very difficult [8–10]. This study reported our results of the use of MFC-free vascularized bone grafting in the management of persistent non-union in scaphoid fractures after failed fixation which provided promising results in our study group.

This reconstruction method showed a good rate of scaphoid union in our group of patients (13 out of 16 showed full union 82%), good functional outcomes with the improvement of ROM and wrist pain, VAS score improved from 6 to 2, Mayo score improved from 45 to 73.

Our study limitations included limited the number of cases, and the absence of comparison between the results of this management technique and other treatment methods, these should be addressed in forthcoming studies.

The early appropriate treatment of scaphoid fractures is proven to provide improvement of patient’s symptoms and prevent complications and wrist arthritis, however, scaphoid fractures are frequently missed and neglected in up to 30% of cases evaluated with plain X-ray only [11].

Revision surgeries for scaphoid nonunion after failed fixation are challenging with questionable results. The reported results in the literature regarding these revision surgeries are highly diverse, with variable rates of the union from 50% to 100%. Such variation could be related to different techniques with different implants and different grafting methods, together with variable postoperative protocols. Vascularized grafts have shown the most favourable results in such revision surgeries in patients without SNAC wrist (Table 2 [6–15]).

**Table 2:** Outcomes of revision surgery for scaphoid nonunion with proximal pole osteonecrosis nonunion treated with MFC-free vascularized graft vascularized bone graft.

Study	Total no.	No. with osteonecrosis + previous surgery	Source of graft	No of united scaphoid	Union Rate (%)
Chang et al. [13]	48	11	Vascularized distal radial	24	50
Doi et al. [14]	10	8	Free vascularized MFC	10	100
Arora et al. [15]	21	21	Free vascularized iliac crest		76
Jones et al. [7]	12	9	Free vascularized MFC	10	100
Tambe et al. [16]	11	11	Vascularized distal radial	6	55
Our study	16	16	Free vascularized MFC	13	81.25

A study including 48 patients recorded a union rate of 82% in the management of persistent scaphoid nonunion after failed previous fixation with the use of vascularized or nonvascularized grafting according to fracture pattern [16]. However, this study did not record the presence of AVN, which plays an important role in successful treatment [17].

Another study by Smith and Cooney included 19 cases with failed scaphoid fixation and persistent nonunion, they reported union in 14 of the cases after using grafts whether vascularised or not. The conclusion is that the success of revision surgery depends on the appropriate choice of graft type according to fracture characteristics. Vascularised bone grafts provide a better outcomes in cases with proximal pole AVN [18].

A systematic review by Moon et al. included 19 studies evaluating revision scaphoid fixation using vascularised bone grafting in 184 cases, the rate of the union in all cases was about 86% [19].

Another study by Chang et al. reported that the use of 1,2 ICSRA pedicle graft provides a good outcome in cases with scaphoid AVN however is not sufficient in restoring the length of scaphoid and carpal bones alignment [12]. A more bulky vascularized graft with adequate cancellous bone and structural support may be needed. In 12 of 15 patients, the use of free iliac crest vascularized grafting was successful in achieving union in patients with AVN [20].

Free vascularized bone graft harvested from the medial femoral condyle was first used in the treatment of non-united metacarpals and forearm fractures [21]. Doi et al. reported the use of MFC vascularised onlay graft in the successful treatment of 10 cases with non-united scaphoids [13]. Also Jones et al. used MFC vascularised interposition structural grafts in the treatment of non-united scaphoids AVN and carpal collapse. Union rates in such 2 studies were 100% in 17 cases [22].

According to these previous results our study reported promising results with the healing of 13 out of 16 scaphoids after the second operation (82%), improvement in carpal height and postoperative S-L angle which reflects the improvement in scaphoid length and alignment. The use of a CT scan is considered to be an important method for management planning. It is useful for the evaluation of fracture healing and carpal degeneration. Such results were comparable to other mentioned studies evaluating such reconstruction techniques.

Using vascularised MCF grafts is found to be successful in the management of persistent scaphoid nonunion after failed previous fixation in presence of AVN with good union rates and clinical improvement. Our study results showed that the restoration of scaphoid bone vasculature and proper alignment of carpal bones using vascularized medial femoral condyle grafts may lead to successful treatment of nonunions after failed previous fixation attempts. This technique may be also useful in patients with scaphoid AVN or carpal collapse. Our study union rate is a bit lower than other studies which shows that a lot of factors could contribute to the union rate such as smoking, age and method of fixation and should be standardized in all studies to get comparable results (Table 2). Long-term follow-up is recommended with a larger study group and careful recording of the morbidity of the donor site for evaluation of the efficacy of this management technique.

## Conclusion

We concluded that the free MFC vascularized bone grafts represent a great choice in the treatment of scaphoid nonunions after previously failed fixation showing improvement in the overall functional outcome with the correction of scaphoid humpback deformity. Patients can expect considerable pain relief and improvement in grip strength without great morbidity. We believe that this is a welcomed addition to the repertoire of treatment modalities addressing scaphoid nonunion.
